# A New Disease or Food Poisoning?

**Published:** 1918-05-11

**Authors:** 


### A NEW DISEASE OR FOOD POISONING?

### The Position Defined and the Public Directed.

A PROCLAMATION of the appearance of a new disease naturally and properly excites popular attention, if only on the general principle that to be forewarned is to be forearmed. The announcement, too, has a certain individual interest, for each man must regard himself as a possible victim, and the more closely he peruses the sensational press the more imperious will this possibility appear to him. There are dramatic qualities in the arrival of a new form of mischief and they readily lend themselves to public presentation. As opposed to all this, the attitude of the medical profession generally is one of frank incredulity. The discoverer himself is convinced, of course, both of the truth of his finding and of its importance. But he appeals to an audience which has heard similar announcements that have come to naught and is disposed to think that the experience will be repeated on the present occasion. Yet there have been exceptions, and hence the truly scientific mind will preserve an impartial balance and will consider whatever evidence can be advanced in support of the thesis. A hindrance to this attitude is the brevity of life. There are so many claims to novelty both as regards disease and regards remedies, that a strict and personal investigation of each claim would invade time to such an extent that many practical affairs would have to be neglected. It indeed becomes sadly certain that the area of secure knowledge and opinion open to the individual is a very limited one, and that outside this he has either to bow to a more or less indefinite authority or to accept an attitude of agnosticism. In medicine, particularly, this confession of an open mind or of a limited knowledge is difficult, first because the public expects a confident conclusion, and secondly because there are many situations which demand practical action and cannot wait for the investigation of the doctrine involved. In a word, circumstances exist where probability and not certainty is all that can be obtained, though the natural desire of the sick man is for something more definite, both in regard to diagnosis and in regard to treatment. Medicine in its application to patients is an art, not a creed, and it is generally possible for its exponents to act wisely and firmly, even though vague and hazy in reference to opinion.

### A COMPLEX ISSUE.

An illustration of the general remarks offered in the foregoing paragraph is found in a recent experience as displayed in certain medical and popular publications. One of the leading professional journals published a few weeks ago two papers by responsible physicians recording personal experiences of cases in which diagnosis, in accordance with the accepted canons of the art, was difficult or impossible, and suggesting in a tentative fashion that the patients might possibly be examples of a form of food-poisoning. Promptly other observers announce more or less similar experiences, and a confident label is attached, namely *Botulism* or sausage-poisoning, the term, however, by no means implying that the sausage is the only guilty food. The matter soon extends to the lay press, and does not lose in the telling, and coroners and their juries make unqualified pronouncements thereon. They may be right, but the issue is a complex one and will hardly be resolved by so summary a procedure. At least it will not be resolved in the scientific sense of the term. Yet here we have one example of a practical claim which cannot wait for scientific certainty. If under present circumstances there is some unusual risk, or even the chance of some unusual risk, in the eating of sausages and suchlike foods, it is well that the public be informed thereon, even though the alarm in the end should prove to be unfounded and the scare in time be gathered into the crowded lumber-room of forgotten sensationalisms. Such a position is obviously wiser than a course which, while waiting for scientific proof, would perhaps involve the chance of unnecessary and avoidable poisonings.

### THE END AND THE MEANS.

In the present instance the public proclamation in advance of actual demonstration has the further justification that it may be accompanied by the assurance that there is an easy and ready method of protection in the simple process of efficient cooking. No one is harmed unless perchance in some measure the makers of the foods here in question, and many are at least possibly protected from a danger which otherwise they might have incurred. There are medical voices which speak with contempt and condemnation of the handling of these sacred matters by the lay press, and it must be admitted that some of the incidents which mark such performances are wholly to be deprecated. But the broad result is to the public advantage, and when this is gained some charity must be exercised towards the methods by which the end is obtained. It is very undesirable that the public should be alarmed unnecessarily, but it is still more undesirable that they should run the risk of food-poisoning because they are kept in the dark until a medical doctrine receives scientific proof.

Meanwhile the medical profession, quite properly, reserves judgment and waits for more evidence. There are some conclusions relative to the position which are quite beyond challenge. That poisoning by "tainted" food is a possibility, no one questions, and vomiting and other signs of gastro-intestinal irritation are the evidences of it. Again, it is admitted that poisoning may occur from food which is completely free from all the ordinary suggestions of commencing decomposition, and here it is believed that the condition is due to a definite micro-organism especially likely to be found in

**A NEW DISEASE OR FOOD POISONING?**—*(cont.)*.

sausages, ham, potted meats, and so on. Further, it is probable that the poisoning in such circumstances need not produce irritation of the stomach and intestines, but that toxins produced by the micro-organisms enter the blood and so reach and affect the nervous system. Thus there may be no abdominal pain, no vomiting, no diarrhœa, and therefore no obvious suggestion of food influence. Food causation may be obscured also in consequence of delay in the symptoms. When "tainted" food is taken the stomach and intestines soon revolt. But with food containing the specific micro-organism of sausage-poisoning time must elapse before the toxins betray themselves, and hence a suspicion of any particular article of diet may fail to occur either to the victim or to his medical adviser.

### A Prima Facie Case.

Especially is such an attitude likely to prevail in this country, where sausage poisoning in the sense here considered is a very rare experience. One of the questions proposed just now is whether the new food conditions of the day are not bringing to us new experiences which are relatively common in countries where the uncooked or half-cooked sausage and its kindred are popular. Undoubtedly there are suggestions to this effect in some of the experiences recorded in the medical journals. The mental dulness and irresponsiveness of the patient in association with drooping of the eyelids, paralysis of some of the ocular muscles, and dryness of the mouth and throat undoubtedly recall the classical symptoms of *Botulism*, and hence a *prima facie* case may be allowed. But it may well be doubted whether all the cases which are just now rushed into this category deserve the position. The fashion of the moment offers the opportunity, and advantage is taken of it. In this respect it has to be noted that with scarcely an exception the cases are isolated ones, and not more than one member of the household is attacked, even though several people share a common table. Now, as circumstantial evidence at least, one of the strongest suggestions of food poisoning is that a number of persons who have partaken of the incriminated dish are attacked by the same symptoms. This is indeed the rule when the poisoning is due to "tainted" food, and where, presumably, the pernicious agent is some chemical irritant. But the argument is less impressive when applied to a form of poisoning which depends on the activities of a specific micro-organism, as it is a common experience that while many persons may be exposed to a specific epidemic influence relatively few succumb to the poisoning. Individuals, it is said, differ in the degree of their resistance to such influences, which is little more than saying that as a fact some persons suffer from the epidemic and some escape. It may well be the same in reference to the specific germ of sausage poisoning. At all events, it must be allowed that while the restriction of the cases here in question to single members of the household does oppose the suggestion of food poisoning it is by no means a conclusive event.

Among the latest medical reports, however, is an experience which is hard indeed to reconcile with the sausage-poisoning suggestion. It is that of the occurrence of the "disease" in an infant of three months and entirely breast-fed, the mother of the child being in perfectly good health. The question may, of course, be proposed whether cases marked by stupor and some degree of unconsciousness (carrying a suggestion of the presence of meningitis) and occurring in children, are or are not of the same order as the disturbances recognised in adults and named *Botulism*, at least in the meantime. The only certain proof in any individual case is the discovery of the specific micro-organism either in food which is the object of suspicion or in the patient's excreta; and for reasons which have already been stated there are difficulties in both these aims.

### What the Public Should Remember.

From all this it is evident that in connection with the cases to which reference is here made the medical profession has some hard nuts to crack. That there are just now coming under observation a number of cases in which dulling of the consciousness, even to the extent of coma, is a prominent feature is certain, and some of these have been observed in adults, some in children, and one certainly in a breast-fed infant. Do all these cases belong to one and the same group? Are they due to food poisoning? These are the issues, and doubtless they will be successfully tackled. In the meantime the public may regard with equanimity the medical debate so long only as they remember that such foods as have here been mentioned may quite well imply risk, even though they are quite free from all overt evidences of decomposition, and that this risk may be confidently eliminated by the practice of thorough cooking. Here is the effective defence against *Bacillus botulinus* and all his ways. It is urged by the profession as a practical measure, while the true nature of various cases recorded in the medical journals remains an open question. Wise advice, as was urged at the outset of this article, may readily exist with hesitancy of opinion. There is a plain, practical, sensible course to take, and so long as this is followed the public may leave the doctors to settle the scientific disputes as best they may.

